# Effectiveness and Complications of Percutaneous Needle Tenotomy with a Large Needle for Muscle Contractures: A Cadaver Study

**DOI:** 10.1371/journal.pone.0143495

**Published:** 2015-12-01

**Authors:** Camille Chesnel, François Genêt, Waleed Almangour, Philippe Denormandie, Bernard Parratte, Alexis Schnitzler

**Affiliations:** 1 Department of Physical Medicine and Rehabilitation, Hôpital Raymond Poincaré, AP-HP, CIC-IT 1429, 104 Boulevard Raymond Poincaré, Garches, France; 2 Université Versailles Saint Quentin en Yvelines, « End:icap » U1179 INSERM, UFR des Sciences de la Santé–Simone Veil, Montigny-le-Bretonneux, France; 3 Trainer for the European School of Surgery, 45 Rue des Saints-Pères, Paris, France; 4 Physical Medicine and Rehabilitation Department, Laboratoire d’anatomie, UE 3920, Jean Minjoz Hospital, University of Franche-Comté, 20 rue Ambroise Paré, Besançon, France; 5 Université Versailles Saint Quentin en Yvelines, « Handi-Resp » EA4047, UFR des Sciences de la Santé–Simone Veil, Montigny-le-Bretonneux, France; IRCCS E. Medea, ITALY

## Abstract

**Background:**

Twenty-two percent of institutionalised elderly persons have muscle contractures. Contractures have important functional consequences, rendering hygiene and positioning in bed or in a chair difficult. Medical treatment (such as botulinum toxin injections, physiotherapy or positioning) is not very effective and surgery may be required. Surgery is carried out in the operating theatre, under local or general anaesthesia but is often not possible in fragile patients. Mini-invasive tenotomy could be a useful alternative as it can be carried out in ambulatory care, under local anaesthesia.

**Objective:**

To evaluate the effectiveness of percutaneous needle tenotomy and the risks of damage to adjacent structures in cadavers.

**Method:**

Thirty two doctors who had never practiced the technique (physical medicine and rehabilitation specialists, geriatricians and orthopaedic surgeons) carried out 401 tenotomies on the upper and lower limbs of 8 fresh cadavers. A 16G needle was used percutaneous following location of the tendons. After each tenotomy, a neuro-orthopaedic surgeon and an anatomist dissected the area in order to evaluate the success of the tenotomy and any adjacent lesions which had occurred.

**Results:**

Of the 401 tenotomies, 72% were complete, 24.9% partial and 2.7% failed. Eight adjacent lesions occurred (2%): 4 (1%) in tendons or muscles, 3 (0.7%) in nerves and 1 (0.2%) in a vessel.

**Conclusion:**

This percutaneous needle technique effectively ruptured the desired tendons, with few injuries to adjacent structures. Although this study was carried out on cadavers, the results suggest it is safe to carry out on patients.

## Introduction

Muscle contractures have been defined as an osteo-articular deformity caused by a combination of loss of range of joint motion and an increase in resistance during passive mobilization of the limb [1]. The cause of contractures remains uncertain but, in the elderly, appears to be related to hypertonia (hyperactivity of pyramidal or extrapyramidal origin, spasticity etc.) and muscle shortening. Contractures are common with a prevalence of 61.2% in the institutionalized elderly in Philadelphia [2]. If the contractures limit activities of daily living, they are termed acquired deforming hypertonia (ADH) [3]. In France, 22% of the institutionalized elderly have ADH of at least one muscle [3]. Contractures cause difficulty with hygiene, nursing care, dressing, transfers, positioning in a chair or standing as well as cutaneous lesions (fungal infections and bed sores) [1,3]. Certain chronic conditions such as severe traumatic brain injury, stroke, degenerative cerebral lesions or traumatic spinal cord injury may cause contractures [4–6]. Treatment involves botulinum toxin injections, physiotherapy and positioning, however these methods are not always effective. The only truly effective treatment is surgery, such as tendon lengthening, tenotomy and joint surgery. However, surgery often requires general anesthesia and hospital admission [7–13] which comport risks, particularly for fragile patients such as the very elderly. In order to reduce the risks and provide effective treatment, the physical medicine and rehabilitation and neuro-orthopedic teams of Raymond Poincaré Hospital at Garches, France, developed a technique of percutaneous needle tenotomy under local anesthesia. This mini-invasive technique consists of using the bevel of a large needle to section the tendon causing the deformity. Several studies have already been published on the use of this technique for congenital talipes equinovarus in children, chronic tennis elbow and trigger finger [14–19]. The results of these studies are encouraging, for example, the level of effectiveness of percutaneous needle tenotomy for talipes equinovarus is similar to open surgery for 80% of operated children, and toleration is higher [15]. Currently, only a single case of neurovascular complications has been reported in the literature following Achilles tenotomy [20]. With regard to the treatment of muscle contractures, the technique has so far only been used to lengthen the Achilles tendon in children [14–16]. Further studies are thus required to confirm the effectiveness and safety on other tendons. It is important to verify that the technique does not cause macroscopic injuries to adjacent structures and that it effectively ruptures the desired tendon.

The aim of this study was therefore to evaluate the effectiveness and safety (with regard to adjacent structures) of percutaneous needle tenotomy in cadavers.

## Materials and Methods

Ethical approval was not necessary for this cadaver study. According to the 15^th^ of November 1887 law, each living person can specify what they would like to happen to their body after their death. The Paris Descartes Body Donation Center accepts body donations from people in the Ile-de-France region. These bodies are used for research and teaching anatomy and surgical techniques. The study was conducted within training courses at the European School of Surgery in partnership with the Paris Descartes Body Donation Center.

The study was carried out between July 2013 and August 2014 during 2 training courses in the anatomy laboratory of the European School of Surgery in Paris. The aim of the courses was to familiarize doctors with the indications and technical aspects of percutaneous needle tenotomy under local anesthesia.

Thirty-two doctors (6 orthopedic surgeons, 22 physical medicine and rehabilitation doctors (PMR) and 4 geriatricians) carried out the tenotomies. The indications, techniques and potential risks relating to tendon location were explained by an anatomist (BP), a neuro-orthopedic surgeon (PD) and a PMR doctor (AS). The intervention was explained in the following manner: “The tenotomy must be carried out using a 16G hollow needle. Assistance is required in order to place the tendon under maximal tension. The operator must hold the needle in his/her dominant hand at the distal end (close to the bevel) with the bevel positioned perpendicularly to the axis of the tendon. The contralateral hand hooks the tendon. The operator makes lateral movements with the needle (no movement in the superficial to deep direction). The needle must be changed for each tenotomy”. The doctors were taught on the positioning and handling of the needle using the tendon distal to the pectoralis major muscle. Because this tendon was used for training purposes, the results of the tenotomy were not included in the analysis. A demonstration of each tenotomy was carried out on a cadaver.

The interventions were carried out on 8 fresh cadavers conserved at 3°C. The tenotomies were carried out on 16 upper limbs and 10 lower limbs (5 lower limbs could not be used). The cadavers were positioned in supine lying and the limbs were positioned so as to place maximum tension on the tendons during the intervention.

The doctors were instructed to carry out the following tenotomies: the distal aponeurosis of the biceps brachii at the level of the elbow crease, the distal tendons of the flexor ulnaris and radialis muscles at the wrist, the distal tendons of the flexor digitorum superficialis and profundus at the base of the fingers, the distal tendon of the tibialis posterior muscle at the medial arch of the foot, the distal tendon of the abductor hallux muscle at the base of the big toe, the distal tendons of flexor digitorum longus and brevis at the base of the 2^nd^-5^th^ toes, the distal tendon of the tibialis anterior muscle at the instep, the Achilles tendon, the tendon of the biceps femoris muscle just above its distal insertion, the tendons of the semitendinosus and gracilis muscles in the medial part of the knee crease and the aponeurosis of the tensor fascia lata in the distal part of the ilio-tibial band.

Two evaluators, an anatomist (BP) and an orthopedic surgeon (PD) separately evaluated the result of each tenotomy and sought any lesions in adjacent structures, particularly neuro-vascular, by dissecting the area adjacent to the puncture point. The tenotomy was considered to be successful if the tendon was completely ruptured, as partially successful if the tendon was not completely ruptured and as a failure if the tendon had not been touched.

## Results

The results of each tenotomy are described in Table 1 for the lower limb and in Table 2 for the upper limb.

**Table 1:** Percutaneous needle tenotomies in the lower limb.

Tendons	Tenotomies				Neighboring structures potentially at risk	Lesions
	Complete	Partial	Failure	N		
**Tibialis posterior**	4	1	1	6	Posteriour tibial arteries and veins	0
					Tibial nerve	0
					Flexor digitoum longus tendon	1
**Abductor hallucis**	4	3	1	8	Branch of the plantar metatarsal artery	0
					Branch of the medial plantar nerve	0
					Tendon of the medial head of flexor hallucis brevis	0
**Toe flexors**	93	37	0	130	Proper plantar nerves	2
**Tibialis anterior**	6	3	0	9	Dorsal artery and vein of the foot	0
					Deep peroneal nerve	0
					Extensor hallucis longus tendon	0
**Achilles tendon**	8	2	0	10	Peroneal artery and vein	0
					Posterior tibial artery and vein	0
					Tibial nerve	0
					Plantar nerve	0
					Flexor hallucis longus tendon	0
**Biceps femoris**	7	2	1	10	Common peroneal nerve	0
**Semi-tendinosus (ST) et Gracillis (G)**	7 (5ST + 2G)	4 (1ST + 3G)	5 (2ST + 3G)	16 (8ST + 8G)	Popliteal artery and vein	0
					Tibial nerve	0
					Sartorius muscle	1
					Tendon of semimembranosus	2
**Tensor fascia lata**	8	2	0	10	None	0
**Total**	137	54	8	199	**Vessels**	0
					**Nerves**	2
					**Tendons or muscles**	4
					**Total**	6

**Table 2:** Percutaneous needle tenotomies in the upper limb.

Tendons	Tenotomies				Neighboring structures potentially at risk	Lesions
	Complete	Partial	Failure	N		
**Biceps brachii**	6	9	1	16	Vascular bundle	1
					Musculo-cutaneous nerve	0
					Median nerve	0
**Flexor carpi ulnaris**	14	2	0	16	Ulnar artery and vein	0
					Ulnar nerve	0
**Flexor carpi radialis**	9	1	0	10	Radial artery and vein	0
					Median nerve	0
**Finger flexors**	124	34	2	160	Proper digital nerve	1
**Total**	153	46	3	202	**Vessel**	1
					**Nerve**	1
					**Tendons or muscles**	0
					**Total**	2

A total of 401 tenotomies were carried out. 72.3% (n = 290) were complete, 24.9% (n = 100) were partial and 2.7% (n = 11) failed. There were 8 lesions of adjacent structures (2.0%): 4 (1.0%) in tendons or muscles, 3 (0.7%) in nerves and 1 (0.2%) in a vessel.

The musculotendinous lesions which occurred were: a lesion of the long toe flexor (during tibialis posterior tenotomy), a lesion of the sartorius muscle and two lesions of the semimembranosus muscle (during semitendinosus and gracilis tenotomies). Two proper plantar nerves were damaged during the tenotomies of the toe flexors and one proper digital nerve was damaged during the tenotomies of the finger flexors. The vascular lesion occurred during tenotomy of the biceps brachii.

In the tenotomies which failed, there were no lesions of adjacent structures (Table 3) since the needle did not go beyond the subcutaneous tissues.

**Table 3:** Type of lesion depending on the success of the tenotomy.

Tenotomy	Vascular lesions	Nerve lesions	Tendon or muscle lesions	Total lesions	Proportion of lesions among the tenotomies
**Complete**	0	3	4	7	2.4% (7/290)
**Partial**	1	0	0	1	1.0% (1/100)
**Failure**	0	0	0	0	0% (0/11)
**Total**	1	3	4	8	2.0% (8/401)

## Discussion

The results of this cadaver study showed that percutaneous needle tenotomy is effective, with only 2.7% of failures. There were no lesions of adjacent structures in the tenotomies which failed. Overall, lesions of adjacent structures occurred in only 2% of tenotomies (8 lesions), the majority of which were in neighboring tendons (50% of lesions). Nerve lesions only occurred in the distal branches of the proper digital and plantar nerves and no nerve trunks were sectioned. Lesions of the sartorius or semimembranosus muscles or tendons in a patient would not cause any complications and in fact could help to further reduce knee flexion contractures. These muscles are not specifically targeted during the treatment of knee flexion contractures because of the theoretical risk of damaging the tibial nerve or the popliteal vascular bundle. Two lesions which could have negative consequences in patients occurred: damage to a tendon of the flexor digitorum longus during tenotomy of the tibialis posterior and a vascular lesion during tenotomy of the biceps brachii.

### Justification for percutaneous needle tenotomy

Contractures are a common condition. In institutions for elderly people in Philadelphia, 61.2% of residents have a contracture, while in France only 22% have ADH [2–3]. The difference in prevalence between these two studies is related to the fact that not all muscle contractures limit activities of daily living and are thus not considered as ADH. A similar prevalence has been found in patients following stroke despite the different pathophysiological mechanisms [5]. Eighty-five percent of persons with severe traumatic brain injury have at least one muscle contracture and around 60% of patients with spinal cord injury, one year post injury [4,6].

Muscle contractures can limit activity, particularly when they affect positioning in a chair. Prolonged bed rest can cause further complications [3,21]. Difficulties with nursing care and hygiene (washing, changing and dressing) and pain during mobilization are often reported [2,3,21–25]. Moreover, flexion contractures lead to maceration in the folds, fungal infections, sores and bedsores [3,13,22,23].

The occurrence of ADH in such fragile patients is perceived with fatalism by the doctors involved: only 25.4% of doctors believe that ADH can be reversed [3]. Only 8.8% of medical coordinators and doctors in charge of French geriatric units believe that surgical intervention is possible for the treatment of contractures. Classical surgery is risky because of the duration of the intervention as well as the intervention on the tendons, muscles and bones [7–13]. Moreover, postoperative management involves immobilization, bed rest and external fixators which can cause further complications. Such interventions are therefore mostly carried out in young patients with no major behavioral problems, who are generally in good health. One alternative to this heavy intervention is classical tenotomy using a scalpel, in the operating theatre. This percutaneous technique has been described for athletic pubalgia and children with adductor contractures [26,27]. It is a relatively quick technique which, in some cases, can be carried out under simple sedation and local anaesthesia. Moreover, it can be used for deep muscles such as ilio-psoas. Unfortunately, it more often requires prior anaesthesia consultation, general anaesthesia, admittance to hospital and specialised post-operative follow-up. Even if the cutaneous incision is small, problems may occur with skin healing. Theoretically, the use of a needle rather than a scalpel avoids problems relating to healing and reduces the risk of injury to adjacent structures.

We thus felt that it was pertinent and useful to develop a mini-invasive procedure to treat such contractures. Needle tenotomy, carried out under local anesthesia in an ambulatory setting could be a satisfactory alternative treatment for fragile patients in whom the aim is to facilitate nursing care or positioning in a chair and not to improve functional activities.

Needle tenotomies have been carried out in children with congenital talipes equinovarus with no reports of complications [14–17]. Similarly, the technique has been used to free Dupuytren’s contractures with few unwanted effects (cutaneous fissures in 8–16%, infection in 0.7–2% and rupture of a deeper tendon in 0.05–0.2%) [28,29]. Needle tenotomies have also been carried out under ultrasound guidance for chronic tennis elbow and trigger finger with no reports of complications [18,19].

### In practice

The aim of needle tenotomy is to treat muscle contractures or ADH in order to improve comfort, improve positioning in a chair or bed, reduce pressure points (and facilitate bedsore healing) and facilitate hygiene (access to the perineum and treatment of maceration or fungal infections). The aim should be defined prior to the intervention by drawing up a contract with the patient and/or the family or carers. The technique can be proposed if physiotherapy is ineffective, if positioning in bed or in a chair is problematic or to treat associated hypertonia of pyramidal or extrapyramidal origin. This treatment is indicated in patients who cannot undergo classical surgery and who have no potential for recovery. The patients treated are most often elderly, severely disabled and/or have behavioral problems or are in a semi-conscious or vegetative state or have multiple disabilities. The first interventions carried out by our team yielded promising results and are currently being published [30]. Before proposing such a technique, it was essential to understand its effectiveness and safety. The results of this study demonstrated that the majority of tenotomies, carried out by operators who were new to the technique, were successful. Training is essential before beginning to practice needle tenotomies either in an ambulatory setting or in the operating theatre. Close coordination between orthopedic surgeons, PMR doctors and geriatricians is also very important.

The results of this study suggest that the tenotomies which can be carried out without any major risks following training are the distal tendons of muscles: finger and toe flexors, biceps femoris, semitendinosus and gracilis at the knee, abductor hallucis, the Achilles and tibialis tendons as well as the distal aponeurosis of the tensor fascia lata.

In conclusion, percutaneous needle tenotomy seems to be an effective procedure and the risk of injury to adjacent structures is low. Although this study was carried out on cadavers, the results suggest that it is safe to carry out on patients.

### Limitations of the study

The study was carried out on cadavers and the location of anatomical structures may differ from live patients. Moreover, the cadavers did not have muscle contractures thus, although the tendons were placed under tension by providing traction on the muscles, the intervention was more difficult to carry out. In the presence of contracture, the location, tensioning and hooking of the tendon is more straight-forward. Location can also be facilitated by checking for pulses which is obviously not possible on cadavers. Adductor longus for the hip was not retained in this study because of the difficulty to carry out the tenotomy on cadavers: difficulties to locate (lack of pulse) and place the tendon under tension. Despite this, the rate of failure (2.0%) was very low even though the doctors had never carried out the intervention previously and few of them were surgeons.

## Supporting Information

S1 Movie Digital percutaneous needle tenotomy. (AVI)
